# An ENet Semantic Segmentation Method Combined with Attention Mechanism

**DOI:** 10.1155/2023/6965259

**Published:** 2023-02-22

**Authors:** Wei Bai

**Affiliations:** School of Mathematics and Computer Science, Ningxia Normal University, Guyuan 756000, Ningxia, China

## Abstract

Image semantic segmentation is one of the core tasks for computer vision. It is widely used in fields such as unmanned driving, medical image processing, geographic information systems, and intelligent robots. Aiming at the problem that the existing semantic segmentation algorithm ignores the different channel and location features of the feature map and the simple method when the feature map is fused, this paper designs a semantic segmentation algorithm that combines the attention mechanism. First, dilated convolution is used, and a smaller downsampling factor is used to maintain the resolution of the image and to obtain its detailed information. Secondly, the attention mechanism module is introduced to assign weights to different parts of the feature map, which reduces the accuracy loss. The design feature fusion module assigns weights to the feature maps of different receptive fields obtained by the two paths and merges them together to obtain the final segmentation result. Finally, through experiments, it was verified on the Camvid, Cityscapes, and PASCAL VOC2012 data sets. Mean intersection over union (MIoU) and mean pixel accuracy (MPA) are used as metrics. The method in this paper can make up for the loss of accuracy caused by downsampling while ensuring the receptive field and improving the resolution, which can better guide the model learning. And the proposed feature fusion module can better integrate the features of different receptive fields. Therefore, the proposed method can significantly improve the segmentation performance compared to the traditional method.

## 1. Introduction

Computer vision mainly includes tasks such as classification, detection, and segmentation. The classification task is mainly for the whole picture, the detection task is for the part of the image, and the semantic segmentation aims to give each pixel on the input picture a corresponding semantic label [1]. Semantic segmentation is the basis of image processing, and its segmentation results are often used in subsequent image processing [2]. At the same time, compared with image classification or target detection, semantic segmentation can realize more fine-grained reasoning on images [3–5]. However, traditional image segmentation technology faces the problem of difficult feature extraction and training, and it has a high requirement for artificial feature design [6].

In recent years, artificial intelligence (AI) has developed rapidly, driven by new theoretical technologies such as cloud computing, big data, brain science, the Internet of Things, blockchain, and the strong demand for social and economic development [7, 8].

The Chinese government has successively issued important policy documents such as “Made in China 2025” and “New Generation Artificial Intelligence Development Plan” that constitute the core of China's artificial intelligence strategy. At the same time, the U.S. government has successively released the “National Artificial Intelligence Research and Development Strategic Plan,” “Artificial Intelligence, Automation, and Economy,” “American AI Plan,” and other artificial intelligence development strategies [9, 10]. Artificial intelligence has become a new strategic highland for powerful countries to compete [11].

Artificial intelligence has become a decisive technological force leading the future, and it will strongly affect the country's comprehensive competitiveness, economy, security, and social ethics [12]. These phenomena and measures highlight the opportunities and challenges brought about by the development of artificial intelligence and, at the same time, trigger the world's investment in artificial intelligence.

As a branch with great development potential and influence in the field of artificial intelligence, deep learning has achieved remarkable results in cutting-edge tasks such as computer vision, natural language processing, machine translation, and autonomous driving [13–15]. In many application scenarios, computer vision technology is a very important part such as intelligent driving, face recognition, beauty selfie, product selection and traffic flow control, and other applications [16, 17]. Deep learning has also greatly promoted the development of image semantic segmentation technology in computer vision [18].

On the other hand, a large number of application scenarios require precise and efficient semantic segmentation technology. Society's demand for it is becoming stronger, and it has been widely used. The following application areas are examples:

1. Unmanned driving field. Semantic segmentation is widely used in the field of unmanned driving. Sensor devices such as on-board cameras and optical radars perceive the surrounding environment and then input images into the neural network. The computer can automatically segment and classify the images to accurately locate roads and avoid obstacles such as pedestrians and vehicles.
2. Geographic information system. Through training the neural network, the machine can input satellite remote sensing images to effectively identify bridges, roads, houses, farmland, mountains, rivers, and lakes. Different scenes can be automatically marked.
3. Medical image analysis. In recent years, more and more medical researches apply deep learning to the field of medical diagnosis. In the field of intelligent medicine, semantic segmentation can be applied to tasks such as tumor image segmentation and cardiovascular and cerebrovascular diagnosis. And it is stronger than radiologists in the analysing ability, which can greatly reduce the time required for diagnosis.
4. Smart robot. Intelligent robots need functions such as movement, obstacle avoidance, and recognition. In particular, when performing tasks such as medical care, high-precision detection and recognition, industrial production, and high-speed or complex trajectory movement, the understanding of the scene often needs to reach a pixel-level fineness. Semantic segmentation technology can help intelligent robots deeply understand the surrounding environment.

## 2. Related Work

Semantic segmentation is a basic task in the field of computer vision. It is required to mark each pixel in the image with a category according to the context information and spatial structure information [19]. Image semantic segmentation has important applications in various fields. In the field of health and medical care, semantic segmentation technology can accurately extract lesions from patients' X-ray or B-ultrasound images to improve diagnosis efficiency. In the field of autonomous driving, specific information about obstacles can be obtained through semantic segmentation technology to help cars avoid obstacles. In the security field, semantic segmentation technology can accurately segment dangerous goods from security inspection images, realize the automatic detection of dangerous goods, and improve security inspection efficiency. With the development of deep learning technology and the improvement of graphics processing unit (GPU) computing power, training large-scale, deep-level neural networks have become the mainstream in the field of semantic segmentation.

AlexNet is a proposed deep convolutional neural network model [20]. In the 2012 ILSVRC (ImageNet large-scale visual recognition challenge) competition, AlexNet achieved the first place with a top-5 accuracy rate of 84.6%, while the accuracy rate of the second place was only 73.8%. The emergence of AlexNet has aroused the attention of many scholars and unveiled the curtain of deep learning. The authors of [21] propose fully convolutional networks (FCN) by changing the fully connected layer in the last part of the original classification network VGG16 (visual geometry group 16-layer net) [22] to a convolutional layer. The purpose of combining the semantic information of the deep and coarse network layer with the surface information of the shallow and fine network layer is realized, so as to achieve precise segmentation. The authors of [23] proposed that the semantic segmentation network SegNet uses depooling in the decoder to upsample the generated feature maps, so that some subtle edge information is preserved intact. At the same time, in the feature extraction part, SegNet uses the same full convolutional network as FCN, which greatly reduces the number of parameters. The U-Net proposed in [24] adopts a U-shaped structure, splicing the feature map of the encoder stage to the feature map of the corresponding size in the decoder stage, and fuses the spatial semantic information and the pixel information. The decoder learns the information lost in the downsampling and improves the segmentation effect. The DeepLabV1 proposed in [25] uses hole convolution to reduce the size of the feature map while keeping the resolution unchanged. And at the end of the network, the conditional random field (CRF) model is used to restore the edge information and to achieve accurate positioning. The authors of [26] proposed ENet for tasks with low latency and high speed, which greatly improved the speed of the model. It is 18 times faster than the original network model, 79 times less than FLOPs (floating-point operations per second), and the number of parameters is 79 times less. The accuracy is close to that of the original model. The authors of [27] proposed PSPNet (pyramid scene parsing network), which uses expanded convolution to modify the basic residual network ResNet architecture. After the initial pooling layer, the same resolution is used in the entire encoder network. In addition, PSPNet added an additional loss function to the overall learning and achieved the best results. The authors of [28] proposed a new type of decoder structure. First, the deep features and shallow features are cascaded, and then, through the self-attention mechanism, the final decoded features are obtained, reducing the accuracy loss caused by decoding upsampling. The authors of [29] proposed a new type of semantic segmentation algorithm. The dense layer structure is added to the network, grouped convolution is used to speed up the calculation, and an attention mechanism is added to improve the segmentation effect.

Although many achievements have been made in the field of semantic segmentation, there are still too few fusion methods that directly cascade or add shallow features and deep features. The ignorance of the specificity of different features results in loss of segmentation accuracy. In addition, information is lost in the process from downsampling to upsampling, resulting in a decrease in segmentation accuracy.

In response to the above problems, this paper designs a semantic segmentation algorithm combined with attention mechanism. The spatial information path is used to dilate convolution, and a smaller downsampling multiple is used to maintain the resolution of the image and obtain the detailed information of the image. The attention mechanism module is introduced to assign weights to different parts of the feature map, which reduces the accuracy loss. The design feature fusion module assigns weights to the feature maps of different perception fields obtained by the two paths and merges them together to obtain the final segmentation result.

## 3. Materials and Methods

### 3.1. ENet Network Structure

ENet network is a lightweight image semantic segmentation network proposed by Paszke et al. [30], which can achieve target pixel-level semantic segmentation. It has the characteristics of fewer parameters and a fast calculation speed. At the same time, ENet network also has a certain degree of plasticity. Based on this, this paper optimizes and improves the pruning and convolution of the ENet network. A semantic segmentation algorithm based on the improved ENet network is proposed.

**Figure 1:**
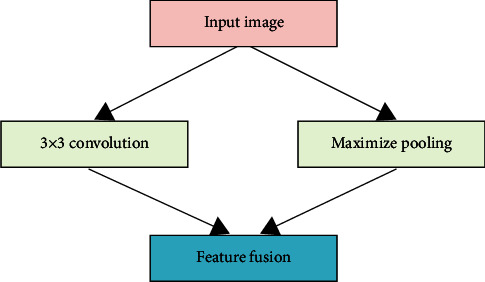
Initialization operation.

The ENet network uses the current general encoder-decoder network structure. This is a lightweight network structure, which has huge advantages in terms of model size and parameter amount. The ENet network changes the previous encoder-decoder symmetry structure. The convolution operation is reduced in the decoder, thereby greatly speeding up the processing speed. The ENet network has an initialization operation for the input image, as shown in Figure 1. Its main purpose is to generate feature maps and merge the feature maps generated by pooling and convolution operations. The convolution operation has a total of 13 3 × 3 filters with a sliding step of 2, and a total of 13 feature maps are obtained. Max Pooling is a noncovered 2 × 2 sliding window to obtain 3 feature maps. Finally, a total of 16 feature maps were obtained after fusion.

**Figure 2:**
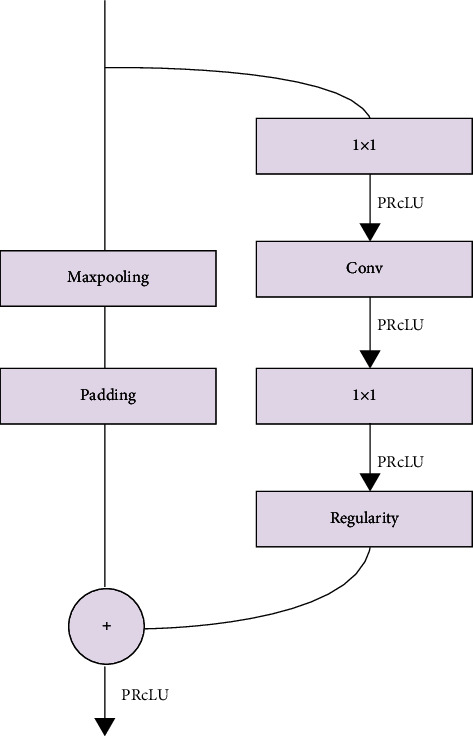
Bottleneck structure.

In addition, a Bottleneck convolution structure is also used in the ENet network, and this module runs through the ENet network. Its main application is in the encoder-decoder, and the concrete structure is shown in Figure 2. Each Bottleneck contains three convolutional layers. In Figure 2, from top to bottom, there are 1 1 × 1 projection mapping for reducing dimensionality, one main convolutional layer, and 1 1 × 1 ascending dimension. And normalization and PReLU activation operations are performed between convolutional layers. Bottleneck is not static and will change according to specific operations. If it is a downsampled Bottleneck, the 1 × 1 projection mapping is replaced by a Max Pooling layer with a kernel size of 2 × 2 and a step size of 2, and it is filled with 0 to match the size of the feature map. Conv is a 3 × 3 conventional convolution, expansion convolution, or full convolution, and sometimes, 1 × 5 and 5 × 1 asymmetric convolution are used instead. Regularization uses spatial dropout to solve the problem of model overfitting.

The overall architecture of the ENet network includes five parts between initialization and the final full convolution. In the first part, there is one downsampled Bottleneck, followed by four ordinary convolutional Bottleneck. In the second part, the first is the maximum pooled Bottleneck, followed by eight ordinary Bottleneck. In the third part, we repeat all operations in the second part except the maximum pooling Bottleneck operation. In the fourth part, there is one upsampled Bottleneck, followed by 2 regular Bottleneck. Since the previous operation has extracted enough feature information, it is necessary to restore the resolution of the image to its original size and output it. Therefore, the fifth part is directly an upsampled Bottleneck followed by an ordinary convolutional Bottleneck. Finally, full convolution outputs the final result of image semantic segmentation. The reason why the expansion convolution module is not used in the fourth and fifth parts is because the coding modules of the first three parts have already segmented the image completely, and there is no need to expand the field of view to extract feature information.

### 3.2. Attention Mechanism Module

**Figure 3:**
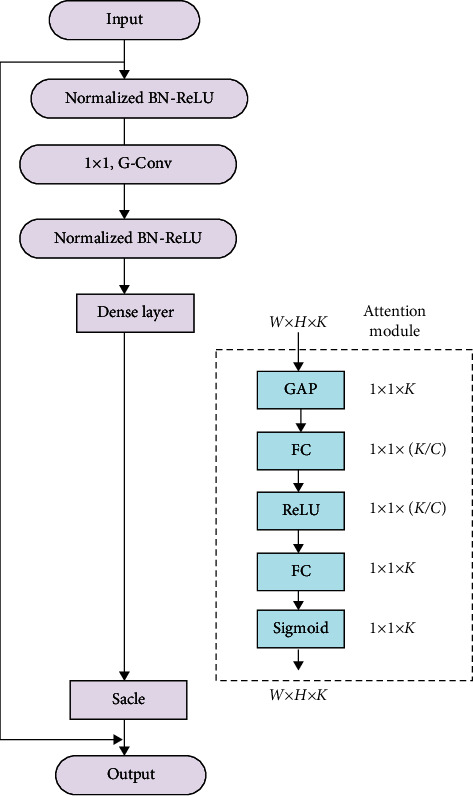
Feature extraction network with attention module.

In order to improve the segmentation speed of the network, this paper uses a lightweight network as the feature extraction network, but there is a certain loss in accuracy. In order to reduce the loss of accuracy, an attention module is added to the network of this article. The attention feature helps to enhance the feature expression of the model, integrate different information, and improve the understanding of the model. This is similar to the attention mechanism of human vision. The human visual attention mechanism is divided into two types: bottom-updata-driven attention mechanism and top-downtask-driven target attention mechanism. Both mechanisms can learn the part needed by the task from a large amount of data. This paper uses a bottom-updata-driven attention mechanism. The attention module starts from the relationship between the feature channels and considers the interdependence factors between the feature channels. Through the self-learning of the network, it effectively suppresses the features that have little influence on the current segmentation effect and enhances the weight of beneficial features. The attention module first performs global average pooling on the feature map of each channel to obtain a 1 × 1 × *K* vector and then performs two FC layer conversions. In order to suppress the complexity of the model, dimensionality reduction and dimensionality increase were performed between the two FC layer conversions, and the Sigmoid and ReLU activation functions were used. The feature extraction network with the attention module is shown in Figure 3.

In the attention module, the global average pooling is first used to shrink the data output from the dense layer from *W* × *H* × *K* to 1 × 1 × *K*. The specific formula is as follows [31]:

(1) $$\begin{array}{c} Z_{c}=F_{sq}\left(u_{c}\right)\frac{1}{H\times W}\overset{H}{\underset{i=1}{\sum }}\overset{W}{\underset{j=1}{\sum }}u_{c}\left(i,j\right)\text{.} \end{array}$$

Among them, *F*_*sq*_ represents the global average pooling function.

Next, two FC operations are performed on the network. *C* represents the dimensionality reduction coefficient, which can be adjusted according to the specific network. The best performance is obtained when *C* = 16 in this experiment, and the formula is as follows:

(2) $$\begin{array}{c} s=F_{ex}\left(z,W\right)=\sigma \left(g\left(z,W\right)\right)=\sigma \left(W_{2}\delta \left(W_{1}z\right)\right)\text{.} \end{array}$$

Among them, *σ* and *δ* represent Sigmoid and ReLU activation functions, *W*_1_, *W*_2_ ∈ *R*^*K*^2^/*C*^.

Then, the scale operation is carried out, and based on the number of channels unchanged, the data are changed to *W* × *H* × *K*.

(3) $$\begin{array}{c} X_{c}=F_{scale}\left(u_{c},s_{c}\right)=u_{c}\cdot s_{c}\text{.} \end{array}$$

Before the network joins the attention module, it takes 42 ms for a forward propagation for input pictures with batches of 256 and 224 × 224. After adding the attention module, it takes 47 ms. After reducing the complexity through dimensionality reduction upgrading, there is still time to increase, but this can be ignored compared to the improvement of segmentation accuracy.

Finally, each layer of the network is connected by a skip connection. The jump connection in this paper is different from DenesNet in two aspects. One is that DenesNet only has connections in the blocks between downsampling, and in this article, there are connections in all layers. The second is in the way of pooling. This article adopts the atrous spatial pyramid pooling (ASPP). ASPP provides a model of multiscale information. It adds void convolutions with different expansion rates on the basis of the spatial pyramid pooling (SPP) to capture a wide range of language territory. Global average pooling (GAP) is combined with image features to increase the global context. A total of four parallel operations, a 1 × 1 convolution, and three 3 × 3 convolutions are included, and the batch normalization is added.

### 3.3. Data Augmentation

This article chooses to conduct comparative experiments on the CamVid data set. The data are collected and annotated by the University of Cambridge. The data set consists of a group of road and street view images, and the resolution size of the data set is 960 *∗* 720. The number of label categories in the data set is uniformly set to 11 categories. The data set consists of three parts, namely training set, validation set, and test set. For the CamVid data set, in order to further expand the number of images to prevent over-fitting, all data preprocessed in the comparative experiment stage in this paper are chosen with the same method as SEGNET to enhance the data set, including Gaussian smoothing, random addition of noise points, color dithering, image rotation, and scaling and other operations. There is no data enhancement for the test set and validation set.

For the Cityscapes data set and PASCALVOC2012 data set, due to the scarcity of data and the limitation of computing resources, we performed the following operations on the data set. First, the five remote sensing images contained in the data set are divided, the first four are used as training sets, and the last one is used as validation sets. Then, we segment the first four images (overlap allowed) into 256 × 256 image blocks. Finally, data enhancement is performed on the obtained image block, including operations such as adding salt and pepper noise, color dithering, image rotation, and scaling.

## 4. Results and Discussion

### 4.1. Introduction to the Experimental Environment and Network Parameter Settings

All experiments in this article are built under the TensorFlow1.9 framework, using cuDNN7.5 kernel calculation, workstation configuration Intel CoreTMi7-6800KCPU@3.4 GHz, GTX1080Ti graphics card, memory 128 GB. The batch size during training is 256, the initial learning rate is set to 0.01, the learning rate attenuation is 10–6, and Nesterov momentum is used. The initialization method is variance scaling, which can adjust the scale according to the weight size, and the activation function uses soft plus. The process of determining the model in this paper is as follows: first, a dense layer is added to the 18-layer feature extraction network. It is found that the network segmentation performance is improved compared to the original 18-layer network, and then, the attention module is added to obtain a better segmentation effect, but the speed cannot meet the real-time requirements. In order to obtain better segmentation performance and faster segmentation speed, a feature extraction network is added layer by layer. We observe the effect of the number of feature extraction network layers on accuracy and speed. Finally, it is determined that the 5-layer feature extraction network structure can maintain the segmentation performance and speed at a high level.

### 4.2. Evaluation Index

Many standards are usually used in image segmentation to measure the accuracy of the algorithm.

Pixel accuracy (PA) is the simplest accuracy measure for semantic segmentation, and it represents the proportion of pixels that are correctly labeled to the total pixels.

(4) $$\begin{array}{c} PA=\frac{\sum_{i=0}^{k}p_{ii}}{\sum_{i=0}^{k}\sum_{j=0}^{k}p_{ij}}\text{,} \end{array}$$

where *p*_*ii*_ represents the correct number of segmentation. *p*_*ij*_ represents the number of pixels that originally belonged to category *i* but were divided into categories *j*. *p*_*ji*_ represents the number of pixels that originally belonged to category *j* but were divided into category *i*. There are *k* + 1 categories (including *k* categories and an empty category or background category).

Mean pixel accuracy (MPA) is a simple improvement of PA. We calculate the proportion of pixels that are correctly segmented in each class and then find the average of all classes.

(5) $$\begin{array}{c} MPA=\frac{1}{k+1}\overset{k}{\underset{i=0}{\sum }}\frac{p_{ii}}{\sum_{j=0}^{k}p_{ij}}\text{.} \end{array}$$

Mean intersection over union (MIOU) is a metric for semantic segmentation technology, which calculates the ratio of intersection and union of two sets. We calculate the IOU within each pixel category and then calculate the average.

(6) $$\begin{array}{c} MIOU=\frac{1}{k+1}\overset{k}{\underset{i=0}{\sum }}\frac{p_{ii}}{\sum_{j=0}^{k}p_{ij}+\sum_{j=0}^{k}p_{ji}-p_{ii}}\text{.} \end{array}$$

### 4.3. CamVid Data set

**Figure 4:**
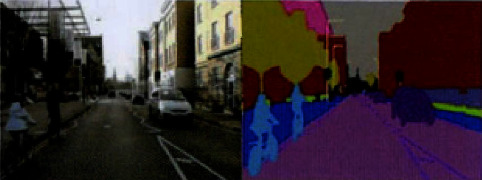
Schematic diagram of annotated images of CamVid data set.

CamVid is the earliest semantic segmentation data set used in the field of autonomous driving. The accuracy of the semantic segmentation result of CamVid data set represents the robustness of the algorithm. At first, five video sequences with a resolution of 960 × 720 pixels were shot on the dashboard of the car, and the shooting angle of view was basically the same as that of the driver. Using image annotation software, 700 images were continuously annotated in the video sequence, including 32 categories such as buildings, trees, traffic lights, sky, roads, pedestrians, motorcycles, cars, and buses. The annotation of an image in the data set is shown in Figure 4.

**Figure 5:**
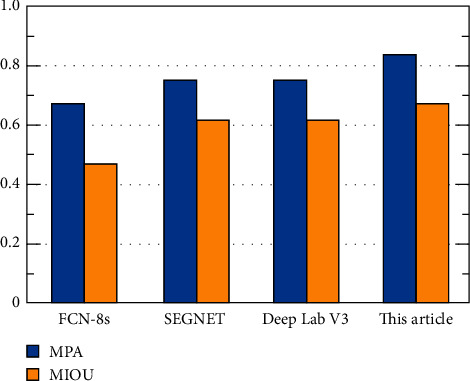
Comparison of experimental results (CamVid).

Under the hardware conditions described in Section 4.1, the CamVid data set is verified, and the models obtained are MPA and MIOU on the CamVid test set, as shown in Figure 5.

Comparing the results in Figure 5, the method proposed in this paper surpasses the original model, FCN model, and DeepLabV3 model in all indicators. And the indicators are slightly higher than the data indicators of DeepLabV3.

**Figure 6:**
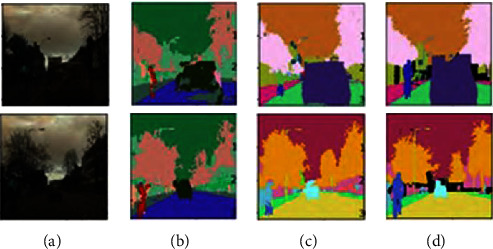
Comparison of segmentation results: (a) original image, (b) traditional model, (c) proposed method, and (d) manual marking.

In order to more intuitively reflect the improvement of pixel category consistency by adding the attention mechanism module structure, we visualized the predicted values of the original SEGNET model and the attention mechanism module structure, as shown in Figure 6. Comparing Figures 6(a) and 6(b), we can see that compared with the traditional model, after adding the attention mechanism module structure, the misdetection of pixel categories contained in the same target is greatly reduced.

### 4.4. Cityscapes Data set

**Figure 7:**
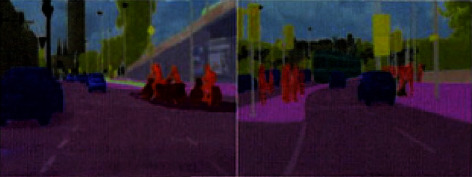
Schematic diagram of annotated images of Cityscapes data set.

Cityscapes is a large-scale data set of 5,000 high-quality images collected from street scenes in 50 different cities. The training set, validation set, and test set of the Cityscapes data set consist of 2975, 500, and 1525 images, respectively, including 19 categories such as ground, building, road signs, nature, sky, people, and vehicles. The data set also provides 20,000 coarsely segmented images for training the performance of the classification network based on weakly supervised learning. The annotation map of the images in this data set is shown in Figure 7.

In order to verify the effectiveness of the improved algorithm, the segmentation results of DeepLabV3 and the proposed algorithm on Cityscapes data set are compared. The comparison results of MIoU values on Cityscapes data set before and after the improvement are shown in Table 1.

It can be seen from Table 1 that the improved algorithm has enhanced the accuracy of most objects' recognition. It shows that the improved network helps to improve the segmentation ability of the network model.

**Figure 8:**
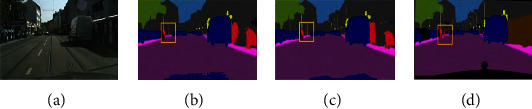
Segmentation results of algorithm on Cityscapes data set: (a) original image, (b) DeeplabV3+, (c) proposed method, and (d) manual marking.

The prediction results of the improved algorithm on the Cityscapes data set are shown in Figure 8. The algorithm in this paper effectively segmented the missing pedestrians in DeeplabV3+ and improved the segmentation ability of small-scale targets.

### 4.5. PASCAL VOC2012 Data set

**Figure 9:**
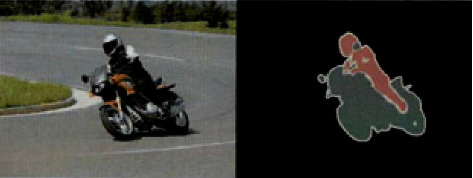
Schematic diagram of annotated images of PASCAL VOC2012 data set.

PASCAL VOC is a challenging competition for the object classification, recognition, and detection in the field of computer vision. It provides standard labeled data sets and evaluation systems for detection algorithms and network learning performance. The training set, validation set, and test set of the PASCAL VOC 2012 data set include 1464, 1449, and 1452 images, respectively, with a total of 21 categories, including humans, animals (birds, cats, cows, dogs, horses, sheep), vehicles (airplanes, bicycles, boats, buses, cars, motorcycles, trains), indoor objects (bottles, chairs, tables, potted plants, Sofa, TV), and background. The annotation of an image of this data set is shown in Figure 9.

**Figure 10:**
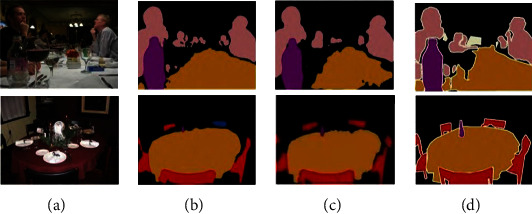
Segmentation results of algorithm on PASCAL VAL 2012 data set. (a) Original image. (b) DeeplabV3+. (c) Proposed method. (d) Manual marketing.

The visualization result of the algorithm on the PASCAL VOC2012 data set is shown in Figure 10.

It can be seen from the first row of Figure 10 that the algorithm in this paper has identified the missing pixels in the DeeplabV3+ network segmentation process, and the prediction result is more accurate. In the result of the second row of segmentation, the DeeplabV3+ network model incorrectly recognizes the chair as another object. The algorithm proposed in this paper avoids this error.

## 5. Conclusion

This paper proposes a semantic segmentation algorithm combined with attention mechanism. Through dilated convolution, a certain resolution is ensured while downsampling obtains a larger receptive field. At the same time, an attention mechanism is added to the network, so that the network can adaptively learn weights and improve the segmentation accuracy. Finally, a feature fusion module is designed to better integrate the features of different receptive fields. The algorithm in this paper is tested on the Camvid, Cityscapes, and PASCAL VOC2012 data sets, and the final results are compared with various algorithms. This method not only guarantees the receptive field and improves the resolution but also significantly improves the segmentation performance. Next, we will further improve the attention mechanism module to explore the specificity and commonalities between different locations and channel characteristics. It is expected to extract more effective feature information, improve the segmentation accuracy of the algorithm for small target objects, and strive for early realization on mobile intelligent devices [32, 33].

## Figures and Tables

**Table 1:** Comparison of MIoU values of the algorithm on the Cityscapes data set.

Classification	Deeplab V3	Improved algorithm
Road	0.913	0.922
Sidewalk	0.845	0.867
Building	0.900	0.912
Wall	0.501	0.518
Sky	0.918	0.932
Train	0.911	0.933
Person	0.942	0.940
Bicycle	0.728	0.786
Fence	0.923	0.923
Traffic light	0.965	0.966
Bus	0.854	0.866

## Data Availability

The data included in this paper are available without any restriction.
